# Concurrent *TSHR* mutations and *DIO2* T92A polymorphism result in abnormal thyroid hormone metabolism

**DOI:** 10.1038/s41598-018-28480-0

**Published:** 2018-07-04

**Authors:** Eunkuk Park, Jaehoon Jung, Osamu Araki, Katsuhiko Tsunekawa, So Young Park, Jeonghyun Kim, Masami Murakami, Seon-Yong Jeong, Sihoon Lee

**Affiliations:** 1 Department of Medical Genetics, Ajou University School of Medicine, Suwon, Republic of Korea; 2 Department of Biomedical Sciences, Ajou University Graduate School of Medicine, Suwon, Republic of Korea; 3 Department of Internal medicine, Gyeongsang Institute of Health Science, Gyeongsang National University School of Medicine and Gyeongsang National University Changwon Hospital, Changwon, Republic of Korea; 4 Department of Clinical Laboratory Medicine, Gunma University Graduate School of Medicine, Maebashi, Japan; 5 Department of Internal Medicine, Cheil General Hospital and Women’s Healthcare Center, Dankook University College of Medicine, Seoul, Republic of Korea; 6 Department of Internal Medicine and Laboratory of Genomics and Translational Medicine, Gachon University School of Medicine, Incheon, Republic of Korea

## Abstract

Deiodinase 2 (DIO2) plays an important role in thyroid hormone metabolism and its regulation. However, molecular mechanism that regulates DIO2 activity remains unclear; only mutaions in selenocysteine insertion sequence binding protein 2 and selenocysteine tranfer RNA (tRNA^[Ser]Sec^) are reported to result in decreased DIO2 activity. Two patients with clinical evidence of abnormal thyroid hormone metabolism were identified and found to have *TSHR* mutations as well as *DIO*2 T92A single nucleotide polymorphism (SNP). Primary-cultured fibroblasts from one patient present a high level of basal DIO2 enzymatic activity, possibly due to compensation by augmented *DIO2* expression. However, this high enzymatic active state yet fails to respond to accelerating TSH. Consequently, *TSHR* mutations along with *DIO2* T92A SNP (“double hit”) may lead to a significant reduction in DIO2 activity stimulated by TSH, and thereby may have clinical relevance in a select population of hypothyroidism patients who might benefit from a T3/T4 combination therapy.

## Introduction

Thyroid hormone metabolism is tightly regulated by the hypothalamic-pituitary-thyroid (HPT) axis through its positive and negative feedback mechanisms. Meanwhile, thyroid hormone regulation at the cellular level remains largely unknown except for several important deiodinases (DIOs). Among them, type 2 iodothyronine deiodinase (DIO2) converts inactive (thyroxine, T4) to “active” thyroid hormone (3,5,3′-triiodothyronine, T3) and plays a significant role as a determinant of the final concentration of T3.

Apart from thyroid-stimulating hormone (thyrotropin, TSH) suppression for the prevention of recurrent thyroid cancer, TSH also has been used as a reference for optimizing levothyroxine (L-T4) dosages for hypothyroidism. L-T4 monotherapy has remained the standard of treatment with the belief that peripheral conversion of L-T4 into T3 is intact^1,2^. However, questions about the efficacy of L-T4 monotherapy still remain since 10% to 15% of patients complain of residual symptoms of hypothyroidism, including neurocognitive dysfunction, poor well-being and physical deterioration. Although there has been inconsistent efficacy across trials with the clinical significance of relatively low T3 in humans not well established, several animal models indicate that maintaining normal T3 levels is a biological priority, and that the elevation in serum T3 through the administration of both L-T4 and liothyronine (L-T3) may have benefit in some patients^3–5^. Recent studies suggest that deiodinase activity may vary from its encoding gene and highlight the possible role for individualized medicine based on DIO single nucleotide polymorphisms (SNPs)^6,7^. Molecular mechanisms on regulating DIO activities enlighten this approach more precisely, not only for the selection of the patients who can benefit to the combination of L-T4 and L-T3, but also for the therapeutic application of thyroid hormones organ- or cell-specifically.

This study first describes patients with a novel form of abnormal thyroid hormone metabolism characterized by substantially decreased DIO2 activity. Mutational analysis reveals that these patients have the *DIO2* T92A SNP and loss-of-function mutations of the *TSHR* gene. Herein, the causal relationship between this combination of genetic alterations and decreased DIO2 activity is described.

## Materials and Methods

### Measurement of hormones in serum and clinical studies

Serum TSH, free T4, and T3, thyroperoxidase (TPO) antibody, thyroglobulin (Tg) antibody and TSH-receptor antibody levels were measured by immunochemiluminescence assays (Cobas, Roche Diagnostics GmbH, Mannheim, Germany). Serum selenium was measured by inductively coupled plasma -mass spectrometry (NexION^®^350D, PerkinElmer Inc., MA, USA). TRH stimulation test was performed as follows: Two ampules (400 μg) of relefact TRH (protirelin^®^, Aventis Pharma Limited, Germany) were administered intravenously at 8:00 AM after overnight fasting. Serum TSH levels were measured before injection of relefact TRH and after 30, 60, 90, 120, 150, and 180 minutes. Informed consent was obtained from participants after study was approved by the Institutional Review Board of Gachon University Gil Medical Center, Incheon, South Korea (GBIRB2013-278) and all research was performed in accordance with relevant guidelines and regulations.

### Molecular genetic studies

Genomic DNA was extracted by standard methods from EDTA blood, and whole exome sequencing was performed. Briefly, a pre-enrichment DNA library was constructed according to the Illumina TrueSeq DNA sample preparation guide (Illumina, Inc., San Diego, CA, USA). Exome enrichment was peformed using Illumina TrueSeq Exome Enrichment probes and streptavidin beads. The enriched exome library was loaded onto flow cells of an Illumina cBot for cluster generation. The flow cells with clusters of the enriched exome libraries were transferred to an Illumina HiSeq. 2000. High-throughput sequencing was then performed for each captured library to ensure that each sample met the desired average sequencing depth of 40-fold. *TSHR* mutations and *DIO2* SNP were confirmed by Sanger sequencing. Genomic DNA was amplified using polymerase chain reaction (PCR) with primers for each exon of the *TSHR* gene^8^ and exon 2 of the *DIO2* gene^9^. The PCR product was sequenced with the forward and reverse primers used for the PCR amplifications using an ABI 3500xL DNA Analyzer (Applied Biosystems, Foster City, CA, USA).

### Cell culture

Fibroblasts were obtained by skin biopsy from patient A and normal controls, and then genotyped: (1) Wild type (WT) (*TSHR* WT + *DIO2* WT), (2) Hetero (*TSHR* WT + heterozygous *DIO2* T92A), (3) Homo (*TSHR* WT + homozygous *DIO2* T92A), and (4) Patient (heterozygous *TSHR* R450H + homozygous *DIO2* T92A). Primary tissue culture was performed by explant technique. Briefly, dissected tissues were finely chopped and rinsed with PBS. The tissues were seeded onto the surface of a tissue culture flask in 1 ml of Dulbecco’s Modified Eagle’s medium (DMEM) medium supplemented with 40% fetal bovine medium volume made up to 5 ml, and then changed weekly until a substantial outgrowth of cells was observed. Cells were then incubated in DMEM medium supplemented with 15% FBS, penicillin (100 U/mL), and streptomycin (100 μg/mL). All cultured cells were incubated in a humidified atmosphere at 37 °C and at 5% CO2. The study used cells with 5–10 passages for all experiments. The C2C12 myoblast progenitor and IMR90 human fibroblast cells were grown in DMEM supplemented with 10% heat-inactivated fetal bovine (FBS), penicillin (100 U/mL), and streptomycin (100 μg/mL). The TαT1 cells were kindly provided by Prof. B. Haugen (University of Colorado), and cultured as described previously^10^. The TαT1 cells required plates coated with Matrigel (Decton Dickison BD) to facilitate adhesion. Briefly, Matrigel was diluted 1:30 in DMEM and allowed to air dry in hood before plating cells. The TαT1 cells were seeded on Matrigel-coated plates and grown in DMEM containing 10% FBS, penicillin (100 U/mL), and streptomycin (100 μg/mL). All cultured cells were incubated in a humidified atmosphere at 37 °C and at 5% CO_2._

### TSH and cAMP Treatment and quantitative RT-PCR

Primary cultured cells (2 × 10^4^ cells/well) were seeded into a 6-well culture tray. After 24 hour incubation, the cells were serum-starved for 18 hours. For TSH treatment, different TSH concentrations (0, 1, and 10 IU) were added for either 3 or 6 hours. For cAMP, cells were treated with 1 mM db-cAMP (Sigma) for 16 hours. Total RNA was extracted from cultured cells using TRIzol reagent (Invitrogen, Carlsbad, USA), following the manufacturer’s instructions (Beckman Coulter, Brea, USA). Extracted RNA was subsequently reverse transcribed using a RevertAid™ H Minus First Strand cDNA Synthesis Kit (Fermentas Inc., Hanover, USA) with oligo(dT)_15–18_ as a random primer. All real time RT-PCR measurements were performed using the ABI Prism 7000 Sequence Detection System (Applied Biosystems, Foster City, USA). All PCR amplifications (40 cycles) were performed in a total volume of 25 μl containing 150 ng of cDNA using an SYBR Green I qPCR kit (TaKaRa, Shiga, Japan), according to the manufacturer’s recommendations. The specific primers for human DIO2 were as follows: 5′-TCCAGTGTGGTGCATGTCTC-3′ and 5′-CTGGCTCGTGAAAGGAGGTC-3′. By normalizing to *Gapdh*, the relative quantification of gene expression was performed using the comparative threshold (Ct) method, as described by the manufacturer (Applied Biosystems). The values were expressed as the fold change over the control. Relative gene expression was displayed as 2^−ΔCt^ (ΔCt = Ct_target gene_ − Ct_Gapdh_). The fold change was calculated as 2^−ΔΔCt^ (ΔΔCt = ΔCt_control_ − Ct_treatment_).

### Western blotting

The TαT1 and IMR90 cells (2 × 10^4^ cells/well) were seeded into a 6-well culture tray. After 24 hour incubation, the cells were serum-starved for 18 hours. Cells were treated with TSH (0, 1, and 10 IU) for 8 hours or T4 (0, 20, and 100 pM) for 16 hours. Samples were lysed at 4 °C in RIPA buffer (150 mM NaCl, 1% Nonidet P-40, 0.5% sodium deoxycholate, 0.1% SDS, and 50 mM Tris buffer, pH 8.0). The lysate were centrifuged at 13000 rpm for 20 min at 4 °C. Supernatant was collected and re-suspended in SDS-PAGE buffer and immediately boiled at 100 °C for 5 minutes. Proteins were analyzed by SDS-PAGE on 12% polyacrylamide gels and electroblotted onto PVDF membrane (polyvinylidine difluoride membrane, Millipore). The membrane blots were blocked with 5% BSA for 1 hour, and then incubated with primary antibodies at 4 °C overnight: anti-DIO2 antibody at a 1:2000 dilution (Abcam), anti-TSHR antibody at a 1:1500 dilution (Abbiotec), and amti-Actin antibody at a 1:2000 dilution (Santa Cruz). After washing with PBS/0.1% Tween-20, the membrane was incubated with secondary antibodies (anti-rabbit or anti-goat antibodies, Santa Cruz) for 1 hour at room temperature and then visualized by the WEST-ZOL plus ECL Western blotting detection system (iNtRON BioTechnology).

### cAMP assay

Primary cultured cells (2 × 10^4^ cells/well) were incubated in 6-well plates for 24 hours and serum-starved for 18 hours. Cells were treated with 0 and 1 IU of TSH for 30 minutes. For the cAMP assay, cells were treated with 0.1 N hydrochloric acid to stop endogenous phosphodiesterase activity. After cell lysis, samples were centrifuged at 600 g at room temperature and supernatant was collected. cAMP was measured using the cAMP direct immunoassay kit (Millpore) following the manufacturer’s instructions. Each experiment was repeated three times.

### Immunostaining of TSHR and DIO2

Pituitary human samples were kindly provided by Prof. S. H. Park (Seoul National University). Tissues were fixed with cold 4% paraformaldehyde in 0.1 M phosphate buffer at pH 7.4 and embedded into paraffin wax. The 4–5 micron of paraffin sections on Fisherbrand Superfrost Plus (Cat #: 12-550-15) slides were loaded into glass slide holders for deparaffinization and rehydration as follows: three times in xylene for 5 minutes, two times in 100% ethanol for 10 minutes, two times in 95% ethanol for 10 minutes, two times in 70% ethanol for 10 minutes and twice in H_2_O for 5 minutes. The rack is transferred into 200 ml of pre-warmed (94~96 °C) target retrieval solution in a glass container in the water bath at 95 °C for 30 minutes and the slides were cooled down on the bench top for 30 minutes. The slides were permeabilized with 0.3% Triton X-100 in PBS for 30 minutes and incubated with blocking buffer containing 10% FBS in PBS for 1 hour. Samples were incubated with primary antibodies; DIO2 rabbit antibody at a 1:1000 dilution (Abcam) and TSHR rabbit antibody at a 1:250 dilution (Abbiotec) in blocking buffer for 2 hours at room temperature. After washing with PBS, secondary antibody FITC goat anti-rabbit IgG antibody at 1:500 dilution (Invitrogen) was incubated for 1 hour at room temperature. Samples were mounted on VECTASHIELD mounting with DAPI (Invitrogen).

### Measurement of Type 2 Iodothyronine Deiodinase Activity

Type 2 iodothyronine deiodinase activity was measured as previously described^11,12^, with minor modifications. Briefly, cells were scraped off, and transferred into 1.5 ml ice-cold buffer [PBS containing 20 mm dithiothreitol (DTT)]. After centrifugation at 3000 rpm for 10 min at 4 ^o^C, the supernatant was discarded. Pellets were sonicated in 120 μl assay buffer (100 mM potassium phosphate, pH 7.0, containing 1 mM EDTA and 20 mM DTT)/tube and were incubated in a total volume of 50 μl with 2 nM of [^125^I]T4, which was purified using LH-20 (Pharmacia Biotech, Uppsala, Sweden) column chromatography on the day of experiment, in the presence of 1 mM PTU for 1 hr at 37 ^o^C. The reaction was terminated by the addition of 100 μl 2% BSA and 800 μl 10% trichloroacetic acid. After centrifugation at 1,500 g for 10 min at 4 ^o^C, the supernatant was applied to a small column packed with AG 50W-X2 resin (bed volume 1 ml; Bio-Rad Laboratories, Hercules, CA) and then eluted with 2 ml of 10% glacial acetic acid. Separated ^125^I was counted with a γ-counter. Nonenzymatic deiodination was corrected by subtracting I^−^ released in control tubes without sonicated samples. The protein concentration was determined by Bradford’s method with BSA as a standard^13^. The deiodinating activity was calculated as fmol I^−^ released/mg protein/hour, after multiplication by a factor of 2 to correct for random labeling at the equivalent 3′ and 5′ positions.

### Statistical analysis

All the experiments were repeated at least three times and the results were presented as the means ± standard deviation (SD), as indicated. Statistical analysis was done using PASW Statistics, version 17.0 (SPSS Inc., Chicago, IL, USA). Statistically significant differences between groups were tested using a Student’s t-test. *P* values < 0.05 were considered significant.

## Results

### Clinical characteristics of patients with abnormal thyroid hormone metabolism characterized by substantially decreased DIO2 activity

**Figure 1:**
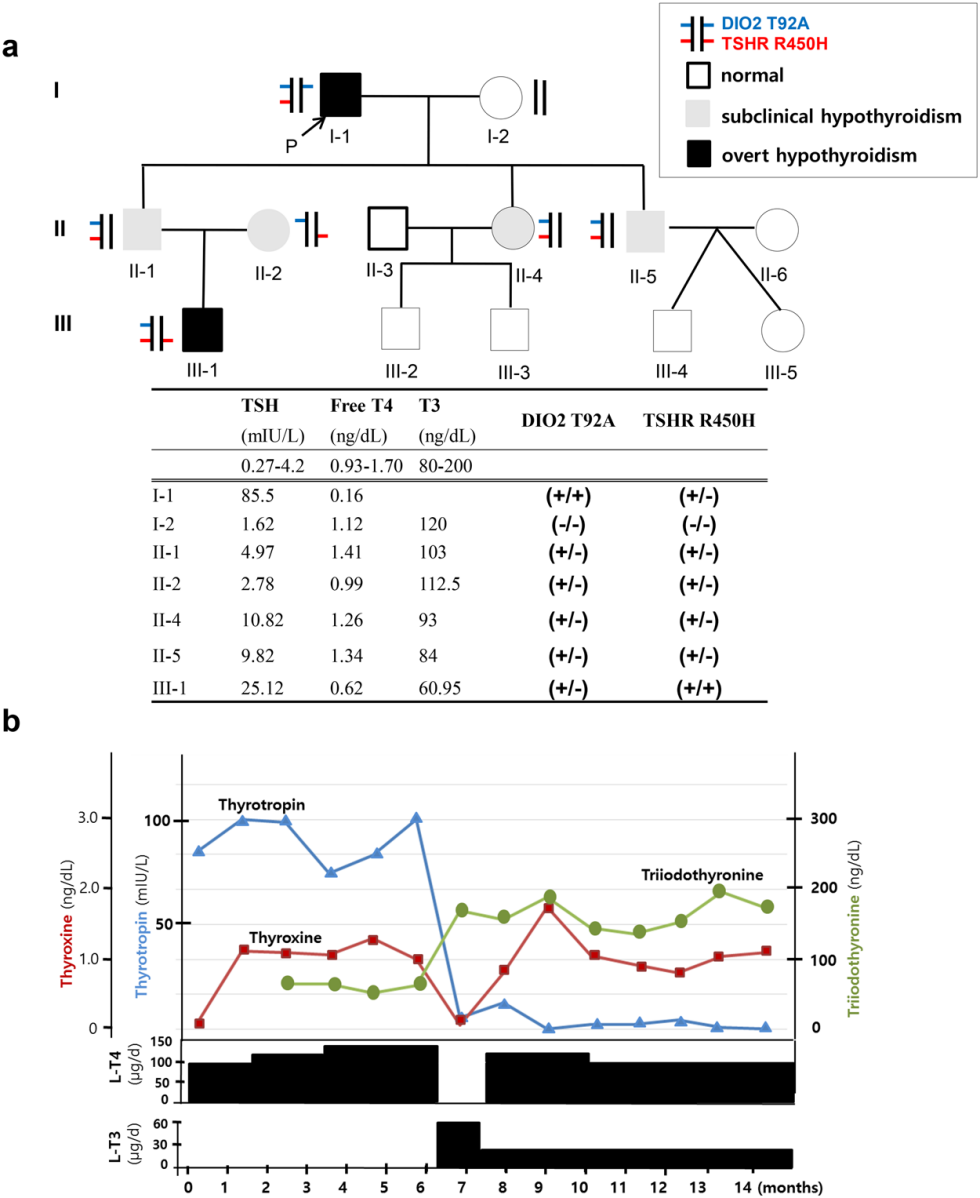
Characteristics of Patient A and his family. **(a)** Pedigree of the Patient A in this study. The letter ‘P’ indicates a proband (Patient A, I-1) of this family. The patients are represented by DIO2 activity and thyroid disease state. The results of *DIO2* and *TSHR* genotypes of the patients and their thyroid functions in this family are also presented. **(b)** Serial thyroid function tests and thyroid hormone dosages of the proband (I-1) are presented. Note that TSH is suppressed only after L-T3 is administered and thyroid function is maintained normal range with combination of L-T4 and L-T3.

A 68-year-old Korean man (I-1 in Fig. 1a; patient A) was referred to the tertiary hospital with abnormal thyroid function tests from a local clinic. The patient had symptoms of anorexia and weight gain for three months. Initial serum TSH was 85.50 mIU/L (0.27–4.20) and free T4 (fT4) was 0.16 ng/dL (0.93–1.70) at local clinic. The patient had hypothyroidism and began to take L-T4 100 μg per day for three months. Although his symptoms related to hypothyroidism improved, his serum TSH level was not suppressed (Fig. 1b). At the hospital, thyroid function and thyroid autoantibody tests were as follows: TSH 98.74 mIU/L, fT4 1.10 ng/dL, T3 64.93 ng/dL (80.00–200.00), Tg antibody 114.8 IU/mL (0.0–115.0), TPO antibody 16.34 IU/mL (0.00–34.00), and TSH-receptor antibody 3.0 IU/L (0.0–1.5). Initially, laboratory error of the immunoassay for serum TSH level was suspected due to heterophile antibody interference. Linearity of serum TSH level was measured following serial dilution, along with serum TSH concentration before and after the addition of a heterophile blocking antibody, and tested by another kit (IMMULITE 2000, Siemens). These three tests for heterophile interference phenomenon were all negative. L-T3 30 μg was administered three times a day instead of L-T4 100 μg per day. Serum TSH level was dramatically decreased to 5.53mIU/L. Thereafter, L-T4 combined with L-T3 was prescribed, and the patient’s thyroid function was maintained normal range up to five years. The patient’s eldest son (II-1) had subclinical hypothyroidism, and his thyroid function was as follows: TSH 4.97 mIU/L, fT4 1.41 ng/dL, T3 103.00 ng/dL, Tg antibody 16.3 IU/mL, TPO antibody 16.4 IU/mL, and TSH-receptor antibody 0.8 IU/L. The daughter-in-law (II-2) had normal thyroid function but positive for autoantibodies for thyroid disease as follows: TSH 2.78 mIU/L, fT4 0.99 ng/dL, T3 112.50 ng/dL, Tg antibody 55.7 IU/mL, TPO antibody 294.2 IU/mL, and TSH-receptor antibody 2.5 IU/L. The patient’s daughter (II-4) and younger son (II-5) both had subclinical hypothyroidism. The patient’s grandson (III-1) had congenital hypothyroidism and was taking L-T4, and his TSH was appropriately suppressed with L-T4, indicating intact DIO2 activity. Neither of the family members, but patient A agreed to provide fibroblast samples.

A 75 year-old Korean woman (patient B) suffered from a thyroid goiter (up to 10 cm in diameter) since her early twenties. Her thyroid function tests revealed extremely high TSH levels, inappropriately high fT4 and relatively low T3 levels; TSH 82 mIU/L, fT4 > 7.70 ng/dL, T3 64 ng/dL without thyrotoxic symptoms. Her goiter decreased in size dramatically when given L-T3 30 μg two times a day, followed by decrease in TSH level to 8.21 mIU/L and increase of T3 level to 94 ng/dL without differences in fT4 level (Table 1). TRH stimulation test revealed that it was less likely due to either RTH or TSH secreting pituitary adenoma for inappropriately increased TSH (Table 2). The patient was strongly suspicious for decreased DIO2 activity but denied the provision of skin samples for evaluation of its activity.

**Table 1:** Serial thyroid function test results and thyroid hormone replacement dosages of Patient B.

	TSH (mIU/L)	Free T4 (ng/dL)	T3 (ng/dL)	Tg Ab (IU/mL)	TPO Ab (IU/mL)	Treatment
	0.27–4.2	0.93–1.70	80–200	0–115	0–34	
0	82	>7.7	64	32.12	12.74	—
1 month	18.64	>7.7	115			L-T3 80ug
2 months	8.21	>7.7	94			L-T3 60ug

TSH, thyroid stimulating hormone; Tg, thyroglobulin; L-T4, levothyroxine; L-T3, liothyronine.

**Table 2:** TRH stimulation test result of Patient B.

	Basal	30 min	60 min	90 min	120 min	150 min	180 min
TSH (mIU/L)	7.76	7.54	11.76	47.30	44.92	55.62	79.79

TSH, thyroid stimulating hormone.

### Genetic analysis of patients A and B identify Concurrent *TSHR* mutations and *DIO2* T92A polymorphism

Nucleotide sequencing of patient A (I-1) revealed to have a homozygous SNP in the *DIO2* gene (c.274 A > G, *DIO2* T92A), which had previously been identified in a certain portion of population as significant SNP^14,15^ and a heterozygous mutation in *TSHR* gene (c.1349 G > A, R450H)^8,16–18^ (data not shown). Interestingly, patients B also had a homozygous *DIO2* T92A together with heterozygous *TSHR* F525S (data not shown). In the family of patient A, I-2 had no genetic alterations in *DIO2* and *TSHR* genes, and II-1, II-2, II-4, and II-5 had heterozygous *DIO2* T92A and *TSHR* R450H. III-1 had heterozygous *DIO2* T92A and homozygous *TSHR* R450H (Fig. 1a). Whole exome sequencing of patient B does not identify any other genetic alterations that could be disease-causing (data not shown).

### Expressions of TSHR and DIO2 are observed in pituitary gland and fibroblast

**Figure 2:**
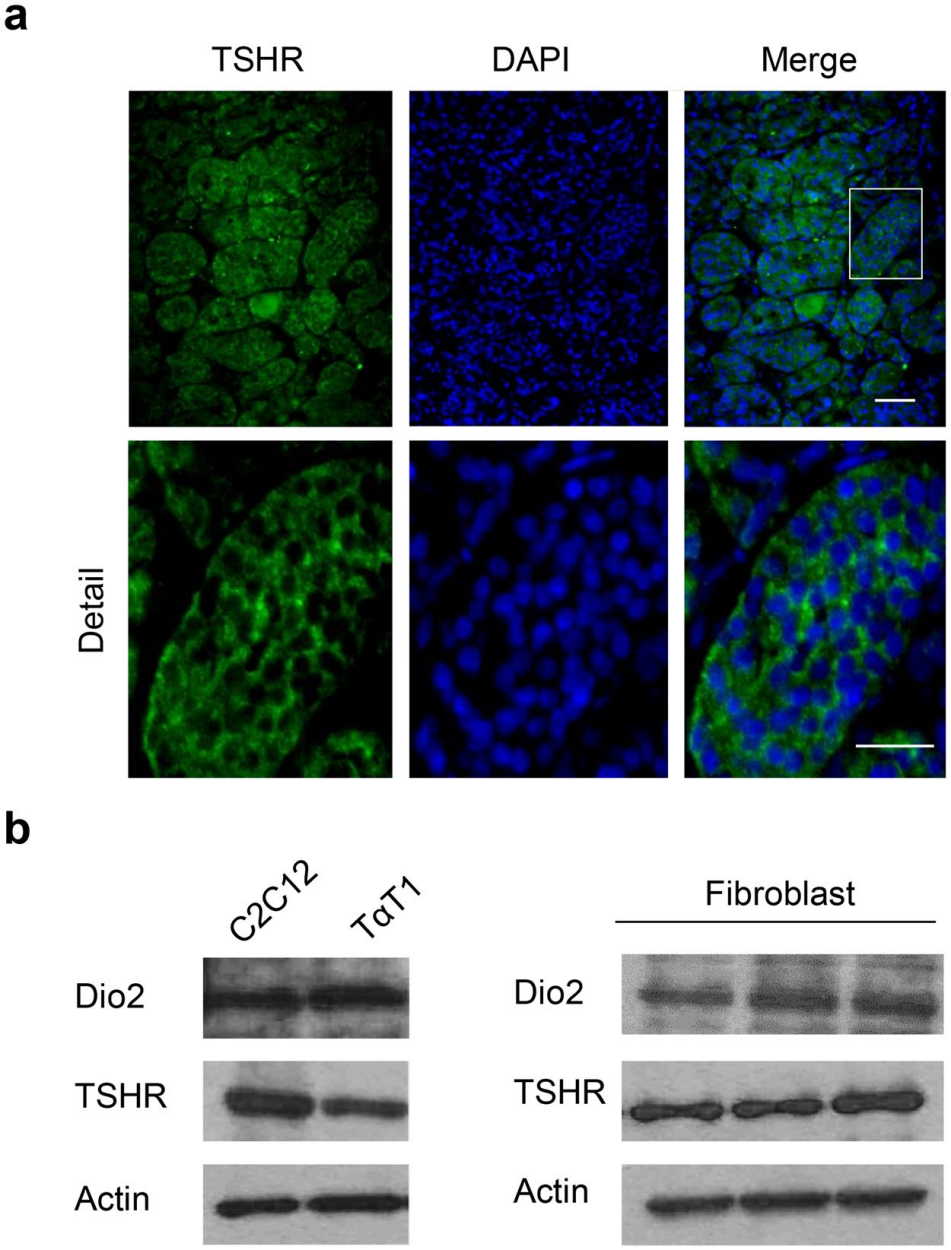
Expression of TSHR and DIO2 in pituitary gland and fibroblast **(a)** (upper panel) Immunocytochemistry results of TSHR in human pituitary tissues. The fixed pituitary tissue is stained with anti-TSHR antibody (green) and mount on VECTASHIELD mounting with DAPI (blue). Scale Bar, 100 μm. (lower panel) Close-up regions indicate localization of TSHR protein on human pituitary. Scale Bar, 50 μm. (**b)** Western blot analysis of DIO2 and TSHR in C2C12 and TαT1 cell lines (left panel) and primary-cultured skin fibroblast cells (right panel). Cells are lysed and analyzed by SDS-PAGE on 12% polyacrylamide gels and electroblotted onto PVDF membrane. Immunoblotting is conducted with the antibodies: anti-DIO2 rabbit antibody at a 1:2000 dilution, anti-TSHR rabbit antibody at a 1:1500 dilution and anti-Actin goat antibody at a 1:2000 dilution. Multiple exposures are shown in the Supplementary Figure 1.

The role of DIO2 in feedback regulation of hypothalamic-pituitary-thyroid (HPT) axis mediated by TSH is well known^19^. In the same context, DIO2 and TSHR are known to be expressed in human pituitary gland and^20–22^ mouse thyrotrope TαT1 cells^23^. Furthermore, DIO2 and TSHR have been reported to be expressed in several types of cells: mouse myoblast C2C12 cells^24^, human osteoblasts^12^, and human fibroblasts^25^. In this study we found that TSHR exists in human pituitary tissues (Fig. 2a) and also confirmed that both DIO2 and TSHR expressed in C2C12 and TαT1 cells and primary-cultured skin fibroblasts (Fig. 2b).

### *TSHR* mutation and *DIO2* variation decrease DIO2 activiy

All patients in this study have heterozygous loss-of-function *TSHR* mutations (R450H and F525S) in addition to homozygous *DIO2* T92A SNP. Biallelic *TSHR* R450H and F525S mutations have been reported to be a cause of congenital hypothyroidism in a few cases^8,16,17,26^. Whether *TSHR* R450H mutations may influence DIO2 activity in patients with biallelic *DIO2* T92A SNP were investigated since his fibroblasts were available and primary-cultured human skin fibroblasts previously used for analysis of thyroid hormone metabolism^25^.

**Figure 3:**
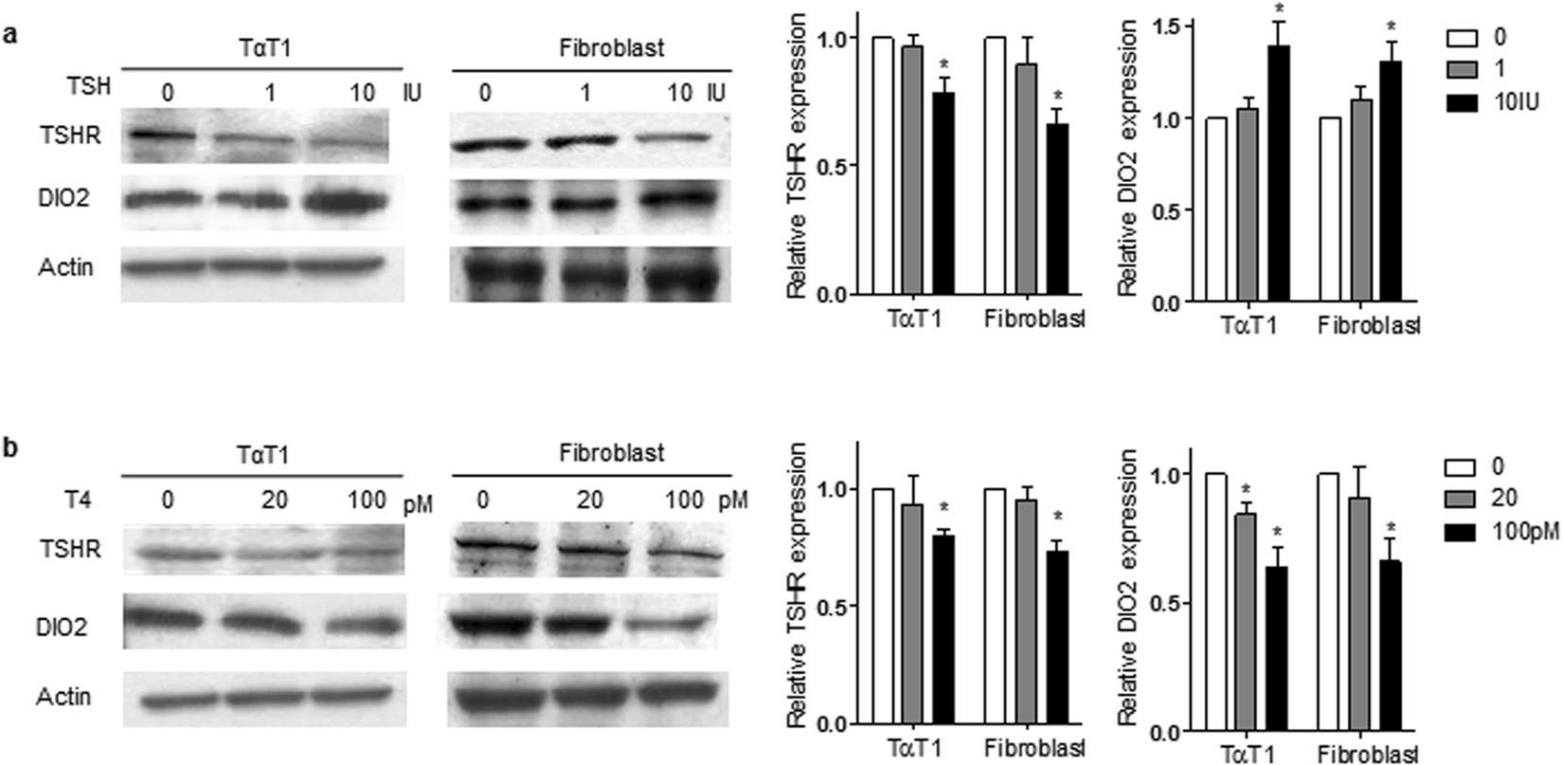
Western blot analysis of TSHR and DIO2 in TαT1 cell line and primary-cultured skin fibroblast cells. Cells are incubated in 6-well plates for 24 h and serum-starved for 18 h, and then incubated with indicated concentrations of either TSH (1 and 10 IU) or L-T4 (20 and 100 pM) for 8 h. Cells are lysed and analyzed by SDS-PAGE on 12% polyacrylamide gels and electroblotted onto PVDF membrane. Immunoblotting is conducted with the antibodies: anti-DIO2 rabbit antibody at a 1:2000 dilution, anti-TSHR rabbit antibody at a 1:1500 dilution and anti-Actin goat antibody at a 1:2000 dilution. **(a)** TSH treatment reveals that TSHR is decreased but DIO2 is significantly increased in 10 IU-treated TαT1 cells and fibroblast. Quantification of band intensity was performed using ImageJ software. *p < 0.05 vs. 0. (**b)** L-T4 treatment reveals that TSHR and DIO2 are decreased dose-dependently in TαT1 cells and fibroblast. Quantification of band intensity was performed using ImageJ software. *p < 0.05 vs. 0. Multiple exposures are shown in the Supplementary Figure 2.

**Figure 4:**
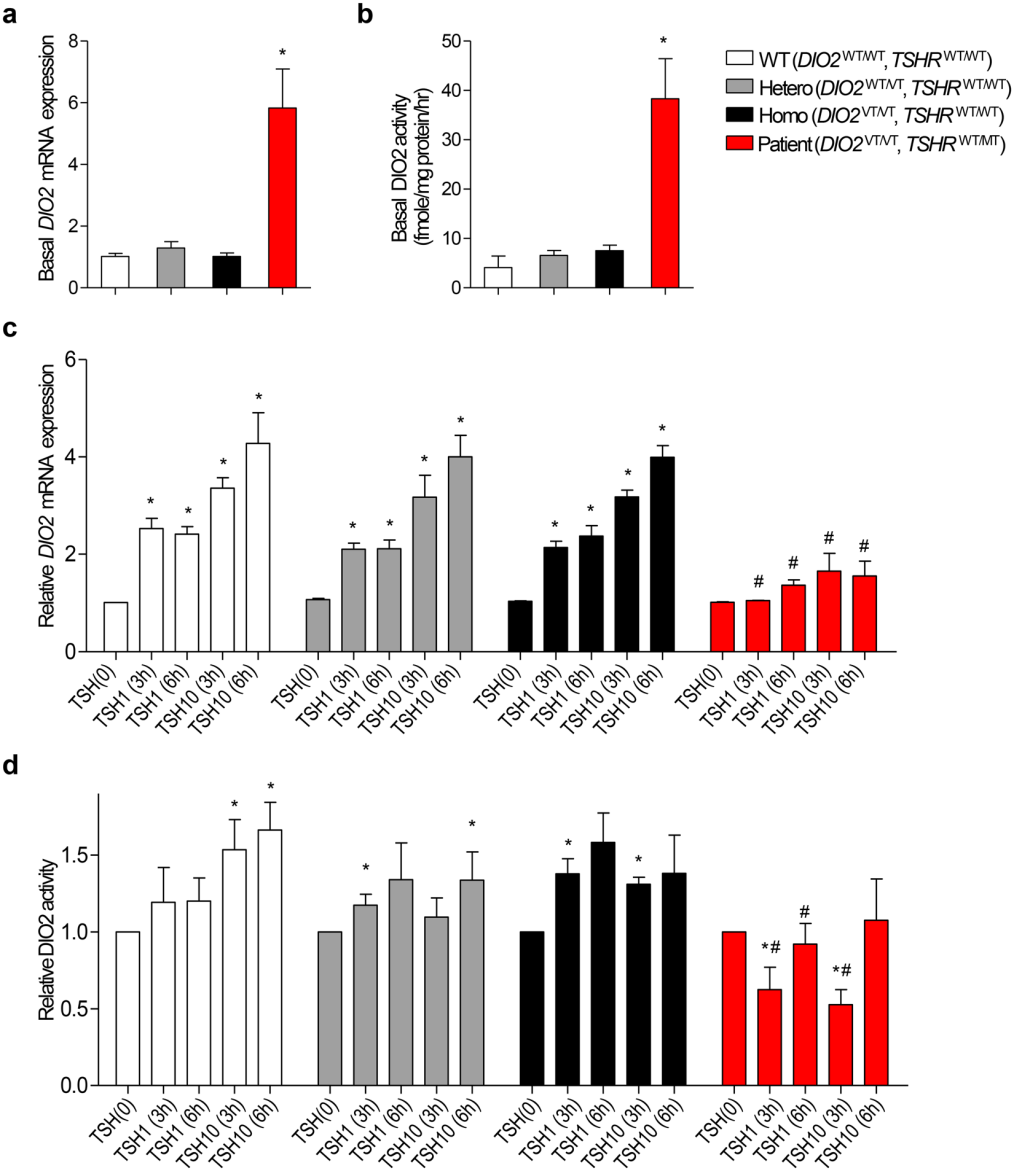
DIO2 expression and activity. **(a**,**b)** Basal levels of DIO2 expression and enzymatic activity in primary-cultured skin fibroblast cells. Cells are from normal controls with wild type *DIO2* genotype (WT), and heterozygous (Hetero) or homozygous (Homo) DIO2 T92A variants, and patient with homozygous DIO2 T92A variant and heterozygous TSHR R450H mutation (Patient). Notably, basal levels of DIO2 expression and activity are significantly higher in the patient’s fibroblast cells than all other tested fibroblast cells. *p < 0.05 vs. WT. (**c)** Relative *DIO2* mRNA expression levels in primary-cultured skin fibroblast cells. The TSH-non-treated cells are used as negative control (0). Total RNA is extracted from the cells and *DIO2* mRNA expression level is analyzed by quantitative real-time RT-PCR. *DIO2* mRNA expressions in WT, Hetero, and Homo are all increased, but no increase is detected in the patient. *p < 0.05 vs. negative control in each tested fibroblast. ^#^p < 0.05 vs. WT in each TSH-treated group. (**d)** Relative DIO2 enzymatic activities in primary-cultured skin fibroblast cells. DIO2 enzymatic activities in WT, Hetero, and Homo are all increased, but no increase is detected in the patient. *p < 0.05 vs. negative control in each tested fibroblast. ^#^p < 0.05 vs. WT in each TSH-treated group.

Primary-cultured fibroblasts were altered with TSHR and DIO2 expression levels in response to TSH stimulation, as reported in TαT1 cells^27^. TSH treatment revealed that TSHR was decreased but DIO2 was increased dose-dependently in fibroblasts and TαT1 cells (Fig. 3a). In contrary, T4 treatment revealed that TSHR and DIO2 were decreased dose-dependently in fibroblasts and TαT1 cells (Fig. 3b). Notably, basal level of DIO2 activity was significantly higher in the patient’s fibroblast cells than all other tested fibroblast cells (Fig. 4a,b). When TSH was treated, *DIO2* mRNA expressions in fibroblast cells of WT, Hetero, and Homo were all increased in time- and dose-dependent manner, but no increase was detected in the patient’s fibroblast cells (Fig. 4c). Furthermore, DIO2 activity also did not increase in the patient’s cells (Fig. 4d).

**Figure 5:**
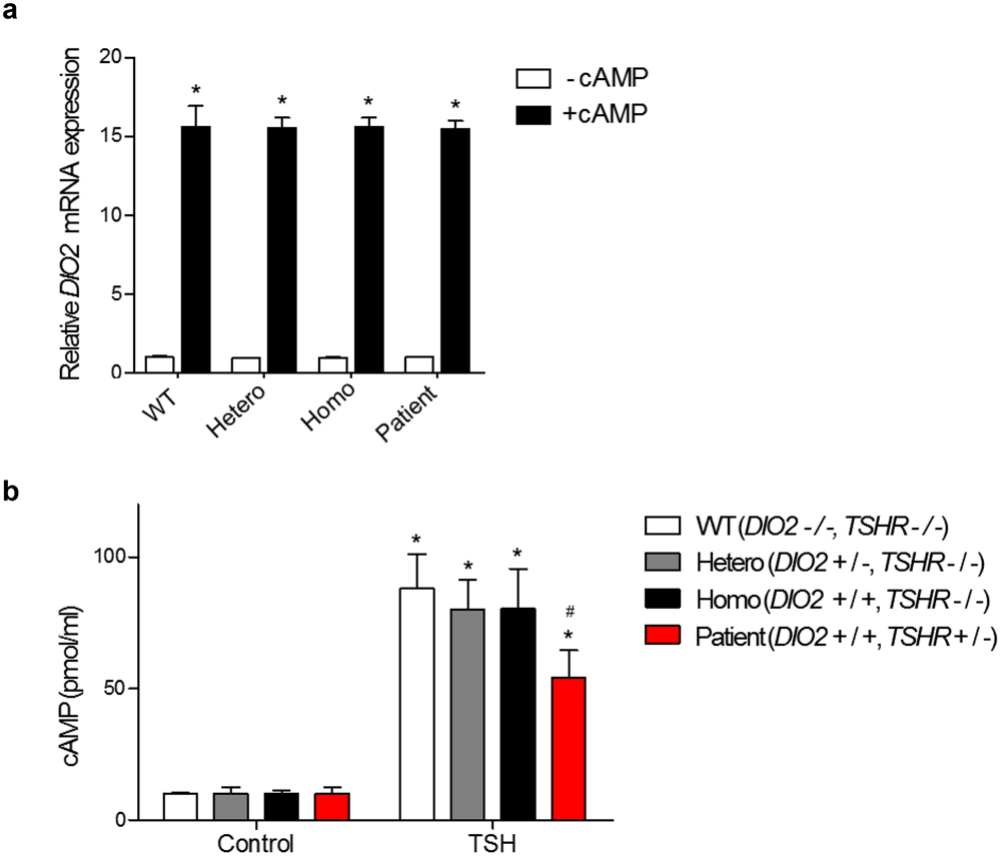
Relative *DIO2* mRNA expression levels in cAMP-treated cells and cAMP assay results in TSH-treated in primary-cultured skin fibroblast cells. **(a)** Cells are treated with 1 mM db-cAMP for 16 h (+cAMP) and total RNA is extracted from the cells and *DIO2* mRNA expression level is analyzed by quantitative real-time RT-PCR. The cAMP-non-treated cells are used as negative control (−cAMP). *DIO2* mRNA expressions in WT, Hetero, Homo and patient are significantly increased by cAMP treatment. *p < 0.05 vs. negative control in each tested fibroblast. (**b)** Cells were treated with 1 IU of TSH for 30 min and then are treated with 0.1 N hydrochloric acid to stop endogenous phosphodiesterase activity. After cell lysis, intracellular cAMP concentration is measured using cAMP direct immunoassay kit. The TSH-non-treated cells are used as negative control. cAMP is increased in all groups, but significantly less increased in the patient. *p < 0.05 vs. negative control in each tested fibroblast. ^#^p < 0.05 vs. WT.

Since DIO2 gene contains a cyclic-AMP (cAMP)-response element^28,29^ in promoter region, cAMP treatment can induce *DIO2* expression even in the absence of TSH. To investigate whether no response of *DIO2* mRNA expression and DIO2 activation with TSH stimulation in patient is caused by *TSHR* mutation, *DIO2* expression changes after treatment of cAMP in the fibroblasts were tested. *DIO2* mRNA expression in patient was significantly increased by cAMP treatment (Fig. 5a). TSH augments cellular cAMP levels^28,29^. Next, the influence of *TSHR* mutation on cAMP production by TSH was tested. When 1 IU of TSH was treated, cAMP was increased in all fibroblasts, but significantly less increased in the patient’s fibroblasts compared to WT, Hetero and Homo (Fig. 5b).

## Discussion

Deiodinases (DIOs), one of the selenoproteins, are essential for life; however, the underlying molecular mechanisms of their regulation remain to be elucidated. For DIO2, high concentration of T4^14,27,30^ and some SNP of *DIO2* gene, especially *DIO2* T92A, have been reported to decrease its enzymatic activity^14^. Although generally believed that *DIO2* T92A is associated with both an impaired baseline psychological well-being on L-T4 and an enhanced response to combination L-T4/L-T3 therapy among hypothyroidism patients^15^, there is still controversy to this assertion because *DIO2* T92A has relatively high prevalence among general populations and all the subjects having this SNP do not show typical characteristics in thyroid function tests. Only a few genetic alterations, which are related to abnormal thyroid hormone metabolism, involve the selenocysteine insertion sequence binding protein 2 (SECISBP2 also known as SBP2) and selenocysteine transfer RNA (tRNA^[Ser]Sec^) mutations^25,31^. There have been a few reports on decreased DIO2 activity arisen from SBP2 mutation worldwide^32–35^.

Since Ishii *et al*. first reported that TSH may induce DIO2 in thyroid gland in 1983^36^, regulation of DIO2 by TSH has been demonstrated in thyroid tissue, brown adipocyte and osteoblasts^11,12,29^. Furthermore, recent studies also show that increased TSH is associated with increased T3 but not T4 as well as T3/T4 in the pediatric age group, suggesting the role of TSH to regulate conversion of T4 to T3 through DIO2^37^. Additionally, the authors have shown that this relationship was also seen in the young adult group. Within the euthyroid range, increasing TSH levels are associated with increasing T3/T4 ratio, indicating TSH enhances preferentially T3 production and proportionally T4 reduction, which results from the DIO2 activity enhanced by TSH^38^.

A previous study reported that cAMP produced by interaction of TSH and TSHR affected the cAMP response element located upstream of promoter region of *DIO2*^28^. The authors’ results also indicate that lack of cAMP production caused by loss-of-function mutation of *TSHR* and *DIO2* T92A SNP cooperatively causes decreased DIO2 enzymatic activity, which is confirmed by experiments of the patient’s dermal fibroblasts. Surprisingly, the patient’s primary-cultured fibroblasts showed exaggerated basal level of DIO2 mRNA expression and enzyme activity, but these fibroblasts had a blunted response to TSH when accerelating doses of TSH was treated, indicating compensatory high enzymatic active state yet fails to respond to TSH.

The *TSHR* R450H mutation, known as a loss-of-function mutation, has uniquely been found only in Korean, Japanese, Taiwanese, and Chinese populations, and were derived from a common ancestral allele^8,16,17,26^. Patients II-1, II-4, and II-5 in family A who have heterozygous R450H had mild subclinical hypothyroidism and patient III-1 who had a homozygous *TSHR* R450H mutation had severe congenital hypothyroidism. The prevalence of *TSHR* R450H mutation is reported as 0.47% in the general Japanease population^18^ and the prevalence of biallelic *TSHR* mutations was estimated to 4.3% in Japanese patients with moderate to severe congenital hypothyroidism^8^. Patient A (I-1) having heterozygous *TSHR* R450H and homozygous *DIO2* T92A showed evident decreased DIO2 activity, but III-1 having homozygous *TSHR* R450H and heterozygous *DIO2* T92A did not show decreased DIO2 activity. Considering age-related deterioration of DIO2 activity which was also referred by recent report showing inconsistency of correlation between TSH and T3/T4 ratio over 40 s and over, DIO2 activity is likely very sensitive to the aging process^39^. In this respect, III-1 needs to be in close monitoring with aging. Other patients (II-4, II5) having heterozygous *TSHR* R450H and heterozygous *DIO2* T92A also need to be evaluated for their DIO2 activity and monitored thereafter.

Another *TSHR* F525S mutation found in patient B was also reported as a loss-of-function mutation^16^. Decreased DIO2 activity in this patient can be explained similarly to patient A. Normalization of TSH in eight congenital hypothyroidism patients by adding a small dose of L-T3 into L-T4 further provides evidence that supports the notion described in this study^40^. More genomic data as well as relevant clinical outcome are needed to confirm this relationship. Lack of direct measured DIO2 activity from patient B and other family members of patient A is a limitation of this study, but patient B is certainly believed to have the decreased DIO2 activity based on their clinical data and presentation.

In conclusion, TSH stimulates DIO2 activity, and *TSHR* mutations and homozygous *DIO2* T92A SNP (“double hit”) result in a novel form of abnormal thyroid hormone metabolism. This study may greatly contribute to the selection of appropriate patients with hypothyroidism who can benefit from L-T3/L-T4 combination treatment, and to provide them with optimized medical care.

## Electronic supplementary material

Supplementary Figure
