# Supplementary material for: Concurrent TSHR mutations and DIO2 T92A polymorphism result in abnormal thyroid hormone metabolism
Source: Sci Rep. 2018 Jul 4;8:10090. doi: 10.1038/s41598-018-28480-0 (PMC6031622; doi:10.1038/s41598-018-28480-0)
Supplement: Supplementary file 1 — Supplementary Figure [file 41598_2018_28480_MOESM1_ESM.pdf]

Supplimentary data

Concurrent *TSHR* mutations and *DIO2* T92A polymorphism result in abnormal thyroid hormone metabolism

Eunkuk Park[1,2*]. **Ph.D.** (eunkuk0815@daum.net)
Jaehoon Jung[3*]. **M.D., Ph.D. (**taesikjung@gmail.com)
Osamu Araki[4]. **M.D. (**oaraki@unma-u.ac.jp)
Katsuhiko Tsunekawa[4]. **M.D. (**ktsune@gunma-u.ac.jp)
So Young Park[5]. **M.D., Ph.D. (**mailbox00@hanmail.net)
Jeonghyun Kim[1,2]. **Ph.D. (**danbi37kjh@daum.net)
Masami Murakami[4]. **M.D., Ph.D. (**mmurakam@gunma-u.ac.jp)
Seon-Yong Jeong[1,2**]. **Ph.D. (**jeongsy@ajou.ac.kr)
Sihoon Lee[6**]. **M.D., Ph.D. (**shleemd@gachon.ac.kr.)

[1]Department of Medical Genetics, Ajou University School of Medicine, Suwon, Republic of Korea;
[2]Department of Biomedical Sciences, Ajou University Graduate School of Medicine, Suwon, Republic of Korea;
[3]Department of Internal medicine, Gyeongsang Institute of Health Science, Gyeongsang National University School of Medicine and Gyeongsang National University Changwon Hospital, Changwon, Republic of Korea;
[4]Department of Clinical Laboratory Medicine, Gunma University Graduate School of Medicine, Maebashi, Japan;
[5]Department of Internal Medicine, Cheil General Hospital and Women's Healthcare Center, Dankook University College of Medicine, Seoul, Republic of Korea;
[6]Department of Internal Medicine and Laboratory of Genomics and Translational Medicine, Gachon University School of Medicine, Incheon, Republic of Korea.

*E.P and J.J contributed equally to this work.

** Addresses correspondence to: **Sihoon Lee, M.D., Ph.D.** Associate Professor. Department of Internal Medicine, Gachon University School of Medicine. 774-21 Namdongdae-ro, Namdong-gu, Incheon 21565, Korea. Tel: +82-32-458-2662, Fax: +82-32-460-2381, E-mail: shleemd@gachon.ac.kr.

Seon-Yong Jeong, Ph.D. Associate Professor. Department of Medical Genetics, Ajou University School of Medicine. 164 Worldcup-ro, Yeongtong-gu, Suwon 16499, Korea. Tel: +82-31-219-4520. Fax: +82-31-219-4521. E-mail: jeongsy@ajou.ac.kr.

Supplementary Fig. S1

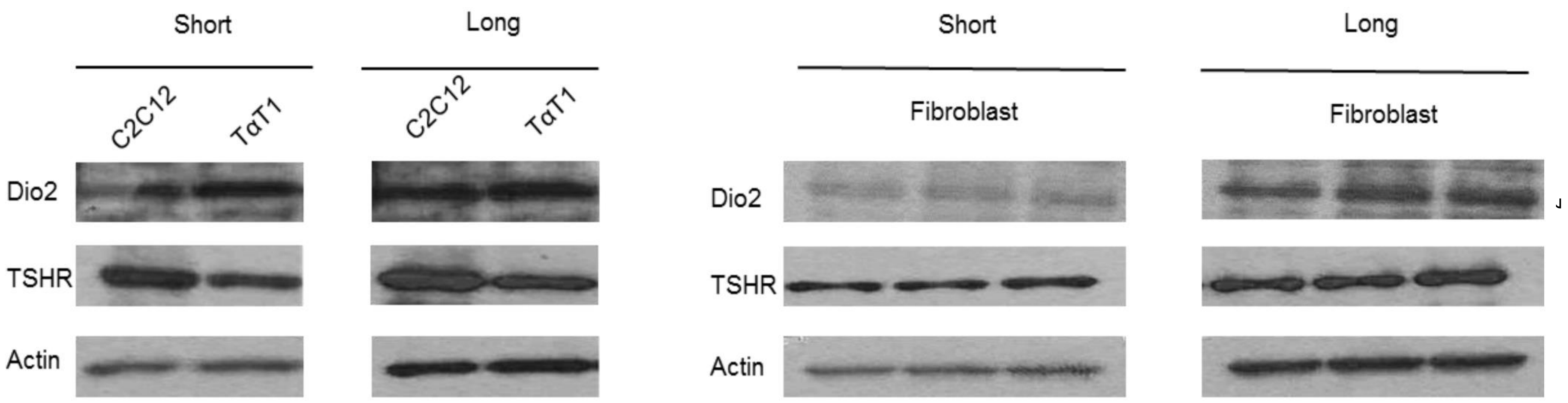

Multiple exposures of Western blot analysis of DIO2 and TSHR in C2C12 and TαT1 cell lines (left panel) and primary-cultured skin fibroblast cells (right panel).

Supplementary Fig. S2

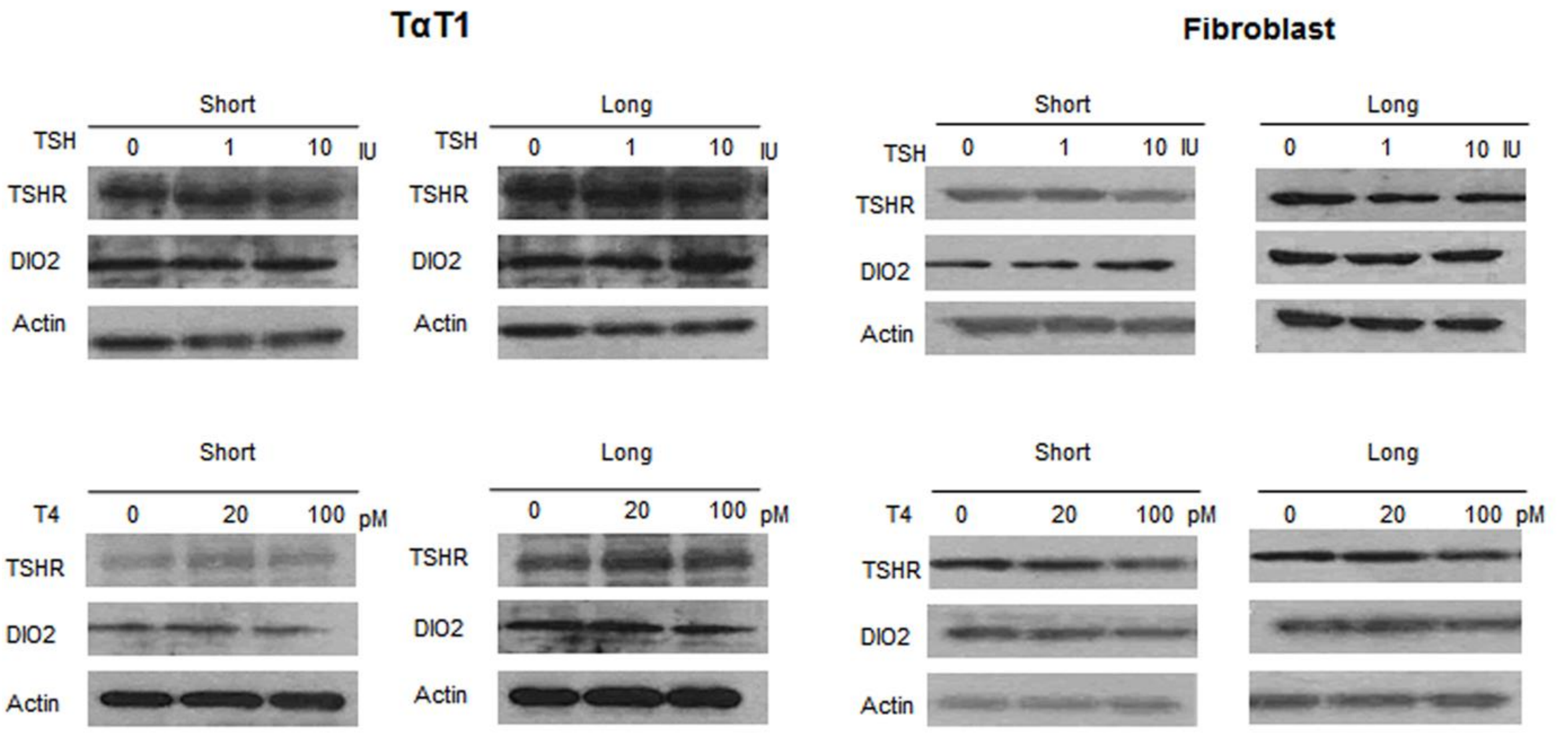

Multiple Western blot analysis of **TSHR and DIO2 in TαT1 cell line and primary-cultured skin fibroblast cells incubated with indicated concentrations of either TSH (1 and 10 IU) or L-T4 (20 and 100 pM) for 8 h.**
